# Detection of antibodies against influenza A viruses in cattle

**DOI:** 10.1128/jvi.02138-24

**Published:** 2025-03-25

**Authors:** Yuekun Lang, Lei Shi, Sawrab Roy, Dipali Gupta, Chao Dai, Muhammad Afnan Khalid, Michael Z. Zhang, Shuping Zhang, Xiu-Feng Wan, Richard Webby, Wenjun Ma

**Affiliations:** 1 Department of Veterinary Pathobiology, College of Veterinary Medicine, University of Missouri 219018 , Columbia, Missouri, USA; 2 Department of Molecular Microbiology & Immunology, School of Medicine, University of Missouri 219013 , Columbia, Missouri, USA; 3 MU Center for Influenza and Emerging Infectious Diseases, University of Missouri 14716 , Columbia, Missouri, USA; 4 Veterinary Medical Diagnostic Laboratory, College of Veterinary Medicine, University of Missouri 70735 , Columbia, Missouri, USA; 5 Department of Electrical Engineering & Computer Science, College of Engineering, University of Missouri 199679 , Columbia, Missouri, USA; 6 Bond Life Sciences Center, University of Missouri 14716 , Columbia, Missouri, USA; 7 Department of Host-Microbe Interactions, St. Jude Children’s Research Hospital, Memphis, Tennessee, USA; St. Jude Children's Research Hospital, Memphis, Tennessee, USA

**Keywords:** IAV infection, cattle, surveillance, ELISA antibody, HI antibody

## Abstract

Unexpected outbreaks caused by the H5N1 highly pathogenic avian influenza virus (HPAIV) in dairy cows in the United States (US) have raised significant veterinary and public health concerns. When and how the H5N1 HPAIV was introduced into dairy cows and the broader epidemiology of influenza A virus (IAV) infections in cattle in the US remain unclear. Herein, we performed a retrospective study to screen more than 1,700 cattle serum samples collected from different bovine breeds in the US from January 2023 to May 2024 using an enzyme-linked immunosorbent assay (ELISA) targeting the nucleoprotein (NP) to detect IAV infections, and the positive samples were further tested by hemagglutination inhibition (HI) assay. Results showed that 586 of 1,724 samples (33.99%) from 15 US states were seropositive by the NP ELISA assay, including 78 samples collected in 2024 and 508 samples collected in 2023. Moreover, the HI assay revealed that 45 of these ELISA-positive samples were positive to human seasonal H1N1 and H3N2 and swine H3N2 and H1N2 viruses, and some were positive to two or three tested IAVs. Surprisingly, none of these ELISA-positive samples were HI positive for the circulating bovine H5N1 strain. Our results demonstrate that IAVs other than H5N1 can infect cattle, infections are not limited to dairy cows, and that bovine infections with swine and human IAVs have occurred prior to the H5N1 outbreaks. All results highlight the value in monitoring IAV epidemiology in cattle, as the viruses might adapt to cattle and/or reassort with the currently circulating H5N1 HPAIV, increasing risk to humans.

**IMPORTANCE:**

Influenza A virus (IAV) is an important zoonotic pathogen that can infect different species. Although cattle were not historically considered vulnerable to IAV infections, an unexpected outbreak caused by H5N1 highly pathogenic avian influenza virus in dairy cows in the United States (US) in early 2024 has raised significant concerns. When and how the virus was introduced into dairy cows and the wider impact of IAV infections in cattle in the US remain unclear. Our retrospective serological screen provided evidence of human and swine H1 and H3 IAV infections in different cattle breeds in addition to dairy cows, although no H5N1 infection was detected. Our results underline the necessity to monitor IAV epidemiology in cattle, as reassortment of IAVs from different species may occur in cattle, generating novel viruses that pose threats to public and animal health.

## INTRODUCTION

Cattle are not considered a natural and susceptible host of influenza A viruses (IAVs), although evidence of IAV infection has been reported in dairy cattle with sporadic milk drop syndrome (1, 2). Recent outbreaks of bovine influenza in dairy cows caused by an H5N1 highly pathogenic avian influenza virus (HPAIV) in the United States (US) have raised significant concerns for human and animal health. The H5N1 HPAIV responsible for the current bovine outbreaks phylogenetically belongs to the 2.3.4.4b hemagglutinin (HA) clade that caused high mortalities in wild birds and domestic poultry in the US (3, 4). From March 2024, H5N1 2.3.4.4b clade virus was detected in two Texas dairy farms and two Kansas dairy farms (5–7), and as of 8 November 2024, the H5N1 HPIAV has been detected in 473 dairy cattle herds in 15 states in the US (8), suggesting that efficient transmission has occurred. Contaminated milking equipment and dairy cattle movements are believed to be the major routes of virus spread among dairy cows (9, 10). Infected cows show clinical signs, such as decreased lactation, reduced appetite, lethargy, fever, and dehydration, and some have to be euthanized due to severe disease (11). Noticeably, a large quantity of virus was detected in milk from lactating cows (12) and 53 human infections have been confirmed in the US, mostly related to outbreaks in milk cows and poultry (13). Adaptation of the virus to more efficient cattle-to-cattle and cattle-to-human transmission will substantially elevate concern for animal and public health. Therefore, it is urgent to perform additional surveillance and experimental research of H5N1 infection of susceptible animal species, including cattle, to fully understand its ecology, transmission route(s), and disease pathogenesis in order to prevent future outbreaks and protect animal and public health.

IAV is an important zoonotic pathogen that can infect avian and mammalian species, including humans (14, 15). IAVs are responsible for influenza pandemics, and annual IAV epidemics cause 250 to 500 million human infections, resulting in 250,000 to 500,000 fatalities worldwide. IAVs belong to the *Orthomyxoviridae* family, have negative-sense single-stranded RNA genomes with eight gene segments, and evolve rapidly through antigenic drift and antigenic shift. To date, there have been 19 HA and 11 neuraminidase (NA) antigenically distinct subtypes identified (16, 17); both H17N10 and H18N11 subtypes were detected in bats, while the other subtypes have been found in waterfowl and shore birds (14, 15). Not all subtypes of IAVs can effectively infect mammalian species, although HPAIV H5 and H7 subtypes occasionally cross species barriers to infect humans, resulting in a high fatality (18). As of 27 September 2024, the H5 HPAIV has caused more than 900 confirmed human infections worldwide, with a case-fatality rate of approximately 50% (19). The H5N1 HPAIV has caused outbreaks in domestic poultry in the US (20) and evolved into 10 HA phylogenetic clades with some further divided into different lineages (21, 22). The clade 2.3.4.4 H5 HPAIVs have been spread by migratory wild birds worldwide and reassorted with local low pathogenic avian influenza viruses in North America, resulting in H5N1, H5N2, and H5N8 HPAIVs (23, 24) responsible for outbreaks in 2014/2015 in the US (25). Since 2021, the H5 clade 2.3.4.4b HPAIVs have caused extensive mortality in wild birds and poultry in the US (26). Importantly, the 2.3.4.4b viruses have been detected in mortality events in marine mammals in Europe and America (27–30) and have been detected in other wild mammals (31). These facts indicate that many mammalian species, such as seals, fox, and bear, are susceptible to the H5 clade 2.3.4.4b HPAIVs, although they likely obtained the virus through contact with infected birds.

Although the outbreaks of H5N1 HPAI in dairy cows were first detected in March 2024, the extent of previous IAV infections in bovines in the US is unknown. In this study, we used an enzyme-linked immunosorbent assay (ELISA) targeting the IAV nucleoprotein (NP) to test 1,724 cattle serum samples collected in 15 US states since January 2023; positive samples were further tested by the hemagglutination inhibition (HI) assay to identify antibodies against H5N1, human seasonal H1N1 and H3N2, and swine H3N2 and H1N2 IAVs. The results showed that 586 beef and dairy cattle samples were ELISA-positive. Seropositivity to human seasonal and swine H1 and H3 viruses was observed but not to H5N1 HPAIV. Our results indicate that both beef and dairy cattle are susceptible to IAV infections.

## RESULTS

### Bovine serum samples positive for IAVs by the NP ELISA assay

**Fig 1:**
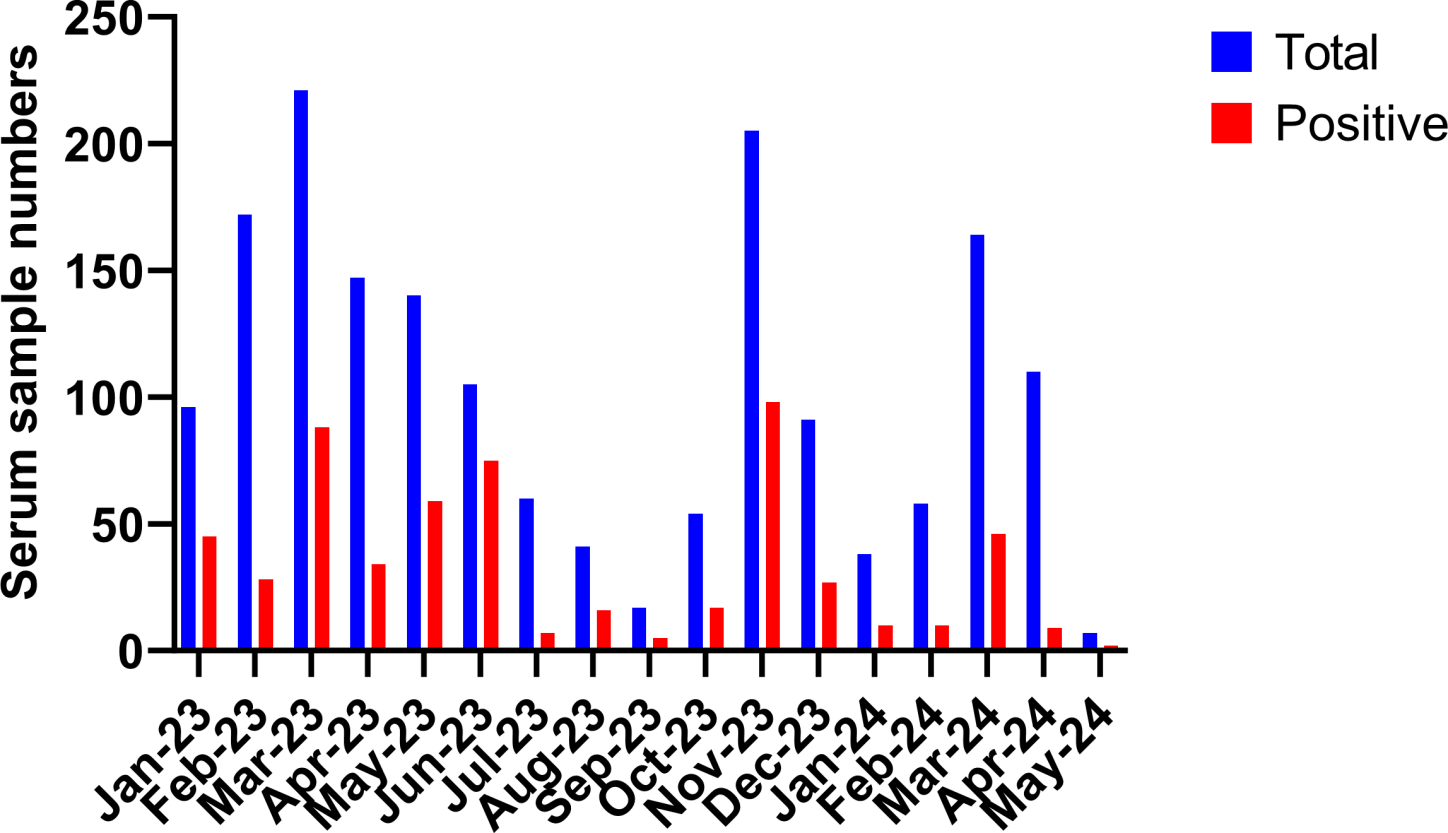
Numbers of bovine serum samples collected in each month from Jan 2023 to May 2024 and seropositive numbers in each month tested by IAV NP ELISA assay.

**Fig 2:**
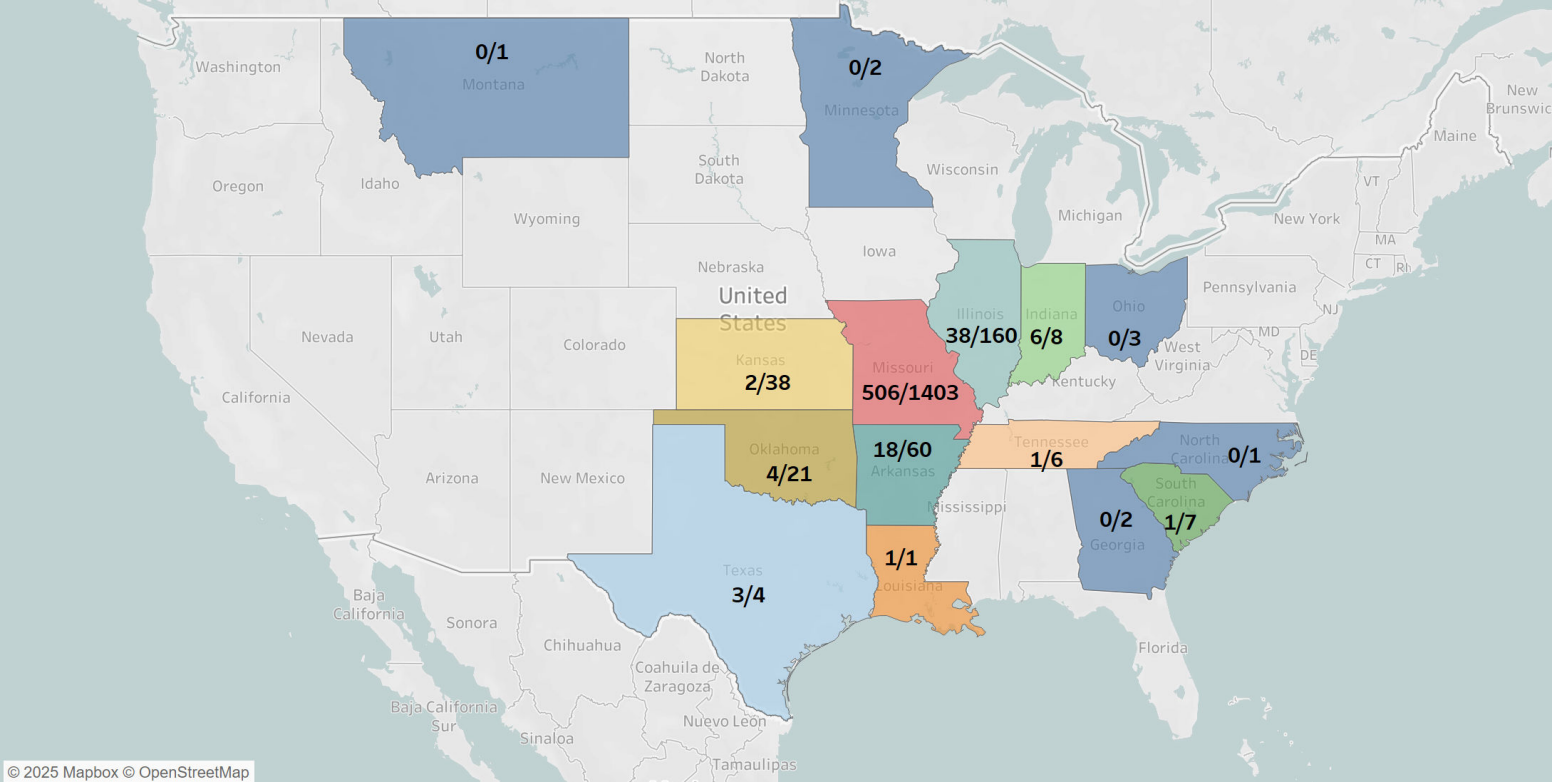
Geographic distribution of bovine serum samples tested and positive sample numbers in different states. Colored areas indicate the states where bovine serum samples were collected. Numbers shown in each state represent the numbers of positive samples/total samples in each state. The map was created using OpenStreet maps via Tableau Public.

**Fig 3:**
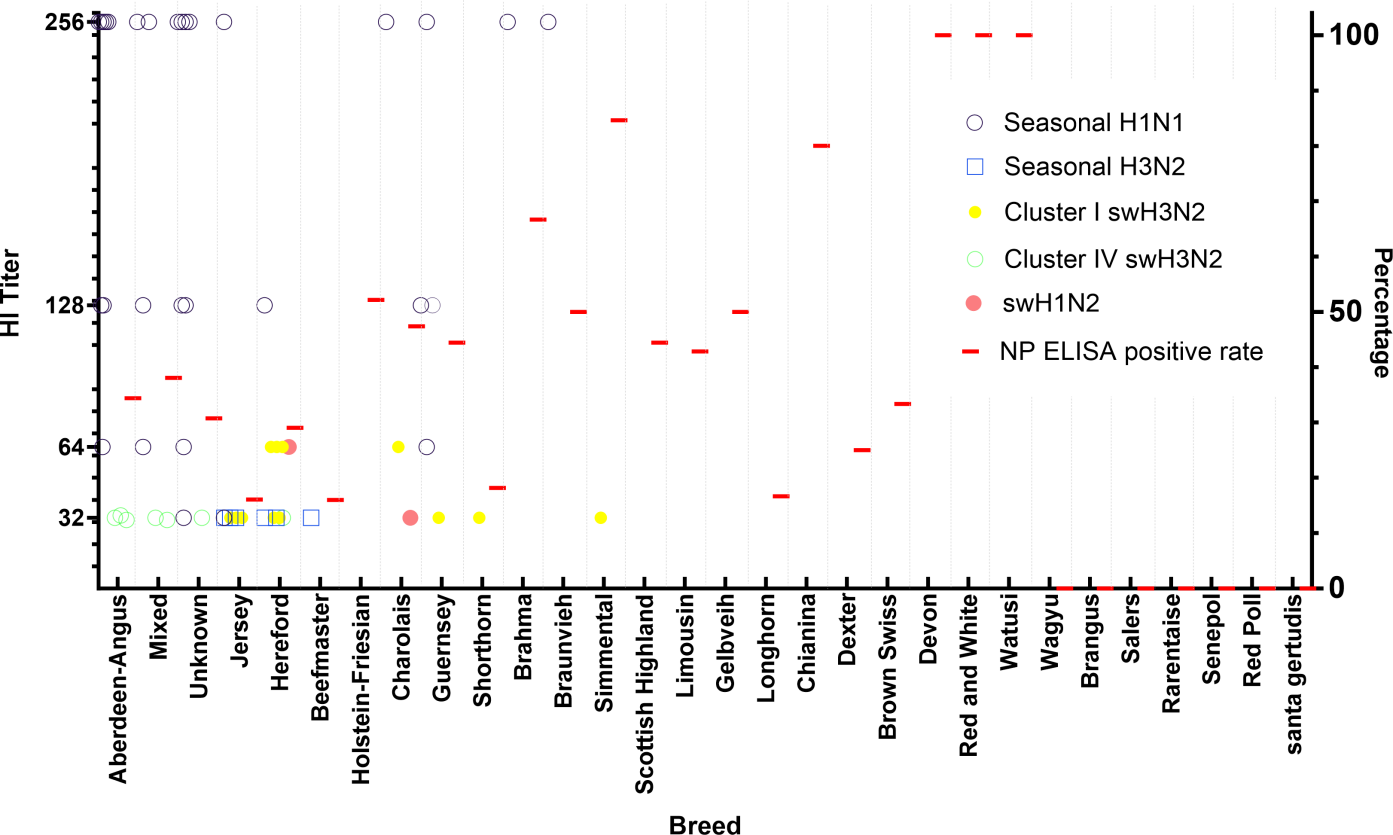
Prevalence of IAV seropositivity in different breeds of cattle. Red colored lines represent NP ELISA-positive rates for each breed. HI titers were measured for NP ELISA-positive samples. For each sample, the reactivity of sera was assessed against six IAVs, including rgH5N1, seasonal huH1N1 and huH3N2, swH3N2 (Clusters I and IV), and swH1N2v. No samples were HI-positive against the rgH5N1. Open circled blue dots denote seropositive samples against seasonal huH1N1. Open blue squares indicate seropositive samples against seasonal huH3N2. Solid yellow dots denote seropositive samples against Cluster I swH3N2. Open green circles represent seropositive samples against Cluster IV swH3N2. Solid red circles denote seropositive samples against swH1N2v.

To determine the prevalence of IAV infections in bovine species, we performed a retrospective serological study through screening 1,724 serum samples using an NP ELISA assay. These samples were collected from at least 30 different breeds of bovines ranging in age from 77 days to 13 years by the Veterinary Medical Diagnostic Laboratory of University of Missouri from January 2023 to May 2024 (Fig. 1) in MO, IL, TX, AR, OK, KS, GA, SC, MN, OH, TN, NC, MT, LA, and IN (Fig. 2 and 3). The majority of samples (1,403) were from MO and collected from 28 breeds of cattle, including dairy cows (Fig. 2). The NP ELISA results showed that 586 out of 1,724 samples (33.99%), representing 23 cattle breeds and 10 states were IAV seropositive (Fig. 2 and 3). Furthermore, we found that the NP seropositive cattle samples were detected every month throughout the year, but the positive rate in winter and spring was higher than that in summer (Fig. 1). In addition, both genders of cattle were susceptible to IAV infection, and no significant difference in the NP-ELISA positive rates was observed among male and female cattle (Table 1). These data indicate that bovine species are susceptible to IAV infections despite the lack of reported detections of the disease in cattle in the US.

**TABLE 1:** Prevalence of IAV seropositivity in different genders of cattle

Gender	NP ELISA positive/total tested (%)	HI titer				
		Seasonal huH1N1	Seasonal huH3N2	Cluster I swH3N2	Clade IV swH3N2	swH1N2v
Male	113/343 (32.84%)	10^*a*^ (32–256)^*b*^	2 (32, 32)	6 (32–64)		1 (32)
Female	366/1078 (33.95%)	15 (32–256)	1 (32)	5 (32–64)	1 (32)	
Unknown	106/303 (34.98%)	6 (64–256)		1 (32)		1 (64)

^*a*^ HI-positive numbers.

^*b*^ HI titer range.

Considering that bovine species are the natural host of influenza D virus (IDV) and the potential cross-reactivity with bovine IDV antibodies, we tested three IDV positive cattle serum samples collected in our previous studies (32) and randomly selected 15 NP ELISA-positive and five ELISA-negative bovine serum samples by the developed NP ELISA assay. The result revealed that 15 NP ELISA-positive samples were positive, while the remaining samples, including three IDV-positive, one IDV-negative, and five NP ELISA-negative bovine serum samples, were negative (Table 2). To further verify the ELISA assay results, we infected MDCK cells with the CA09 H1N1 virus and performed the IFA assay to test nine NP ELISA-positive and four NP ELISA-negative bovine serum samples. Results showed that fluorescent signals were observed in all nine NP ELISA-positive samples but not from four negative samples (Table 2). These results indicate that the developed NP ELISA assay can detect IAV-seropositive bovine samples and is specific, with no cross-reactivity to bovine IDV antibodies.

**TABLE 2:** Results of NP ELISA and IFA assays to test bovine serum samples

Sample ID	NP ELISA	IFA
#24 (IDV HI titer 1,280)	–	n/d^*a*^
#27 (IDV HI titer 640)	–	n/d
#18 (IDV HI titer 640)	–	n/d
#1 (IDV HI negative)	–	n/d
8364-1	–	–
9732	–	–
8367-2	–	–
23096	+	+
12688-39	+	+
16756	+	+
2280-4	+	+
27003-3	+	+
7749-1	+	+
7718-2	+	+
1382-4	+	+
29794-1	+	+
9942	–	–
10495-2	+	n/d
14925-69	+	n/d
5040-15	+	n/d
32280	+	n/d
31494-1	+	n/d
5097-4	+	n/d
8366-1	–	n/d

^*a*^ n/d, not done.

### Specific subtypes of IAV infections in cattle by HI assay

**Fig 4:**
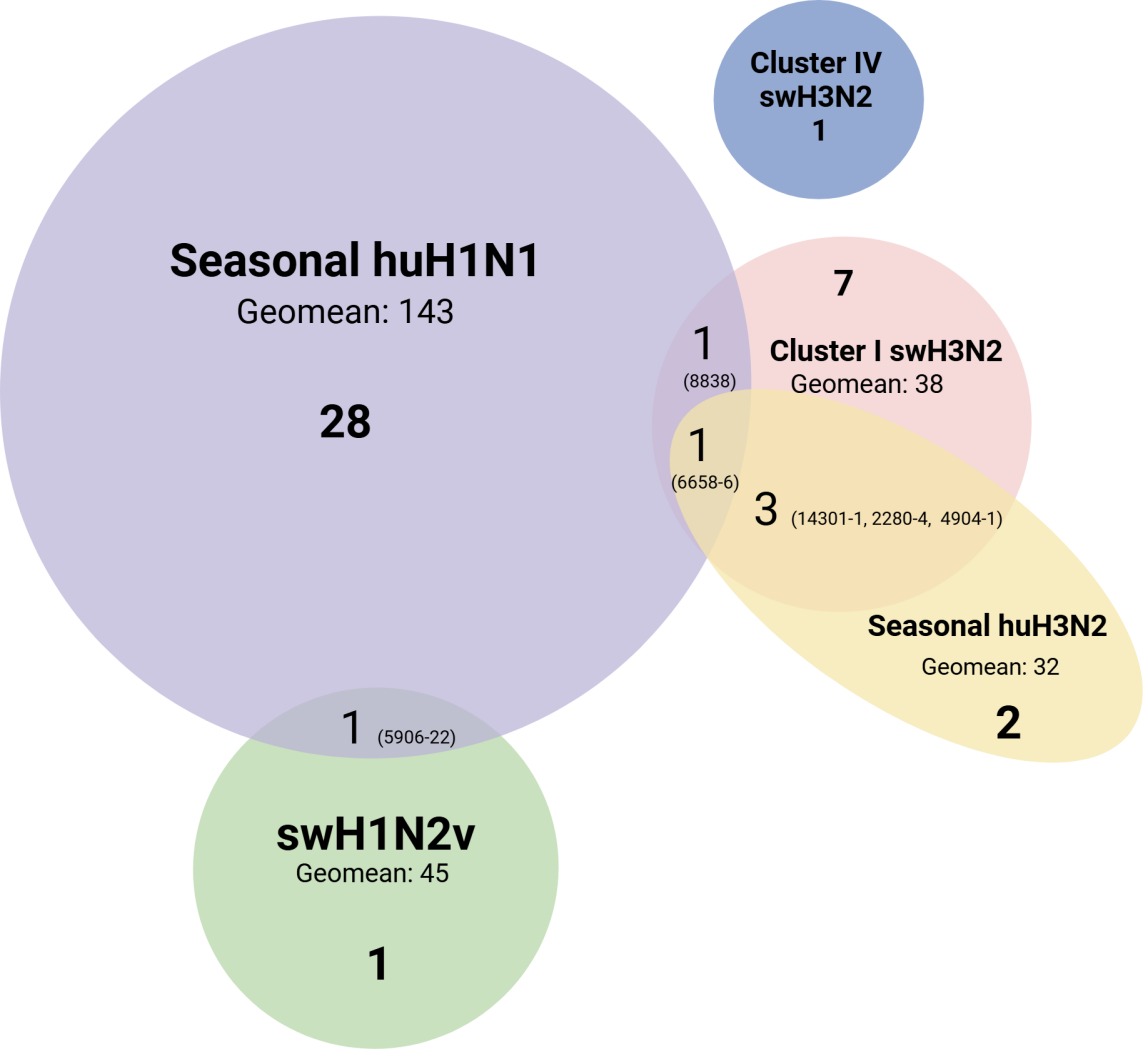
Venn diagram of HI-positive bovine serum samples against human and swine IAVs. Forty-five out of 586 ELISA-positive bovine serum samples were HI-positive to tested human and swine IAVs, including seasonal huH1N1 and huH3N2 viruses, cluster I and IV swH3N2 as well as swH1N2v viruses. None had an HI titer against the tested rgH5N1. The sample IDs were presented in the brackets if samples were dual or triple positive against tested IAV strains, and the geomean of the HI titer against each tested virus was presented.

To determine which subtypes or specific strains of IAVs infected cattle, we further tested 586 NP ELISA-seropositive samples by using the subtype-specific HI assay. Considering the presence of human–cattle and swine–cattle interfaces, human seasonal H1N1 (huH1N1) and H3N2 (huH3N2) viruses, swine cluster I and IV H3N2 (swH3N2), and H1N2 variant (swH1N2v), as well as the rgH5N1 viruses were used for the HI assays. H5N1 HPAIV is responsible for the ongoing cattle outbreaks in the US (6), and both H1 and H3 subtypes of IAVs have been reported to infect cattle (1, 33). Forty-five out of 586 NP-ELISA-positive serum samples were positive for the tested human and swine viruses by the HI assay (Fig. 4). Thirty-six of 45 samples were HI-positive to seasonal huH1N1 and huH3N2 viruses, with titers ranging from 32 to 256 (geometric mean, 121). In these 36 samples, 28 samples were positive for the seasonal huH1N1, five samples were positive for seasonal huH3N2, and three samples were double or triple positive to tested human and swine IAVs (Fig. 4). Fifteen of 45 samples were HI-positive for one of the tested swine IAVs: 12 samples positive for cluster I swH3N2 (TX98), two samples positive for swH1N2v, and one sample positive for clade IV swH3N2 viruses with HI titers ranging from 32 to 64 (geometric mean, 40) (Table 1; Fig. 3). In these 15 samples, six samples were dual or triple positive to tested human and swine IAVs (Fig. 4). None of the 586 NP ELISA-positive samples were positive for the rgH5N1 virus, representing the currently circulating H5N1 HPAIV in cattle.

One serum sample (5906-22) was positive for both swH1N2v and seasonal huH1N1 viruses, and three serum samples (14301-1, 2280-4, and 4904-1) were positive for both Cluster I swH3N2 and seasonal huH3N2 viruses (Fig. 4). In addition, one sample (8838) was positive to both Cluster I swH3N2 and seasonal huH1N1 viruses, while one sample (6658-6) was positive for Cluster I swH3N2 and seasonal huH3N2 and huH1N1 viruses (Fig. 4). These results indicate that both human and swine IAVs can infect cattle, and double or triple IAV infections may generate novel viruses through reassortment in cattle.

**Fig 5:**
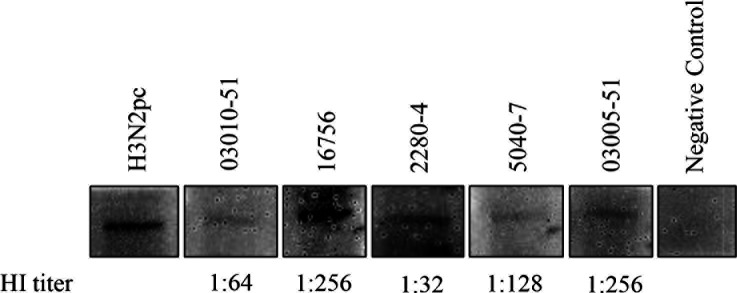
Western blot detection of HI-positive bovine serum samples. Purified NP, HI-positive bovine serum samples (1:50) and an HRP-conjugated mouse anti-bovine IgG (H + L) as the second antibody (1:500) were used for the Western blot assay. A positive serum sample H3N2pc from a calf infected with the TX98 H3N2 at 21 days post-infection and a bovine serum sample negative by HI and ELISA assays served as the positive and negative controls, respectively. The HI titer of each tested positive serum sample was shown.

We randomly selected five available bovine serum samples with HI titers ranging from 32 to 256 for the Western blot assay to detect the purified NP used in the ELISA assay. Results showed that NP was detected by tested HI-positive bovine serum samples (Fig. 5), which further confirmed the findings of the ELISA and HI assays.

## DISCUSSION

Unprecedented outbreaks of bovine influenza caused by the H5N1 HPAIV were first found in Texas and Kansas dairy farms in March 2024 (6), and as of 8 November 2024, have affected more than 470 herds in 15 US states (8). It is very important to understand when and how the H5N1 HPAIV was introduced into cattle herds (9). We performed a retrospective study to screen more than 1,700 cattle serum samples collected from different breeds of cattle of various ages from January 2023 until May 2024 from 15 US states, including Kansas and Texas. Our result revealed a 33.99% IAV seropositive rate based on NP ELISA positivity. This assay is thought to detect all H1-H16 IAV subtypes due to the conserved nature of the NP antigens. Similar results obtained from both IFA and ELISA assays in testing cattle serum samples further verified the developed NP ELISA assay that can detect IAV seropositive samples. IDV has been proposed as a new genus in the *Orthomyxoviridae* family because it is genetically and antigenically distinct from known IAV, influenza B, and C viruses (34). No antibody cross-reactivities were detected between the four influenza virus genera using agar gel immunodiffusion assay (34). Since IDV is prevalent in US bovine herds (34, 35), we tested IDV-positive bovine serum samples using the developed NP ELISA assay to rule out potential cross-reactivity with IDV antibodies in bovine serum samples. IDV-positive cattle serum samples were negative by the NP ELISA assay, further confirming its specificity, although the number of samples tested was limited. Jones-Lang and colleagues have previously found that 58% of bovine sera collected in Minnesota were positive to H1N1 virus by an H1 subtype-specific ELISA assay (36). Another retrospective serological survey of random bovine serum samples submitted in 1999 and 2000 for routine diagnostics at the University of Kentucky Livestock Disease Diagnostic Center revealed that 51% of sera had detectable antibodies to either influenza A/equine/Kentucky/94 (H3N8) or influenza A/swine/Texas/98 (H3N2) (33). We detected influenza NP-positive samples collected from 10 out of 15 states (66.67%). We did not identify positives from MT, MN, OH, NC, and GA, but the sample sizes ranging from one to three of these states were extremely low. Our and other published results demonstrate that US bovine herds have been previously infected by IAVs despite the lack of reported outbreaks, likely due to lack of clinical signs and sustained spread. Surprisingly, none of the 586 NP ELISA-positive samples, including those from Kansas and Texas, were HI-positive to the rgH5N1 virus representative of H5N1 HPAIV responsible for outbreaks in US dairy cows. This could be due to limited samples collected or the fact that most of the samples in this study were from MO, IL, and AR states that have not yet reported H5N1 HPAI cases (8). The results from this study cannot provide answers on when and how the H5N1 HPAIV was introduced into dairy cattle. Therefore, more retrospective serological studies in cattle are needed in the US to address this important question.

Although no H5N1 HPAIV-positive samples were detected, we found that some NP ELISA-positive samples were positive to specific human and swine IAVs by the HI assay, indicating that human–bovine and swine–bovine interfaces play a major role in spreading IAVs to cattle. The seropositive percentages to tested human IAVs were higher than those to tested swine IAVs, indicating that human–bovine interaction is one of the major pathways of IAV infection of cattle. Additionally, HI titers and positivity rates to the human H1N1 virus were significantly higher than those against either tested human H3N2 or swine IAVs, suggesting that the 2009 H1N1pdm virus might be better able to infect bovine species. This is consistent with the fact that the 2009 H1N1pdm virus is able to cross the species barrier and has been detected in pets, turkeys, swine, giant pandas, and wildlife in addition to humans (37–39). Interestingly, IAV seropositive samples could be detected in different breeds of cattle of both genders and of various ages.

We identified several serum samples that were HI-positive to both swine and human IAVs. Considering the potential cross-reaction between seasonal huH1N1 and swH1N2v and seasonal huH3N2 and cluster I swH3N2 viruses, we retested seasonal huH1N1 HI-positive samples against the swH1N2v and cluster I swH3N2 HI-positive samples against the seasonal huH3N2 virus and vice versa. The results confirmed that no cross HI titer was detected in these samples (Table S1), indicating that IAV dual or triple infections in cattle appear to be genuine. Notably, one sample was HI-positive to both cluster I swH3N2 and seasonal huH1N1 viruses, while another sample was positive for cluster I swH3N2, seasonal huH3N2 and huH1N1 viruses. These findings indicate that co-infection with IAVs might occur in some individual cattle. Considering that the H5N1 HPAIV is circulating in dairy cattle herds (8) and cattle can be infected with human and swine IAVs, reassortment of IAVs from different species might happen in cattle (10), generating novel genotypes of H5Nx virus with pandemic potential or at least with increased ability for zoonotic transmission.

In summary, our retrospective serological study demonstrated that IAVs other than H5N1 HPAIV are able to infect different breeds of cattle regardless of their gender and age. Therefore, IAV infection in cattle is likely more complicated than recognized. It is necessary to monitor bovine IAV epidemiology through systematic influenza surveillance to prevent IAV adaptation and potential reassortment that would increase the threat to public health.

## MATERIALS AND METHODS

### Viruses

IAVs including rg-A/American wigeon/South Carolina/22-000345-001/2021 (rgH5N1), human seasonal A/Cambodia/e0826360/2020 (H3N2) and A/Guangdong-Maonan/SWL1536/2019 (H1N1)pdm09, swine influenza A/swine/Texas/4199-2/1998 (H3N2), A/swine/Kansas/11-110529/2011 (H3N2), and A/swine/Kansas/12-156064/2012 (H1N2) were used in this study. These viruses were amplified in 10-day-old chicken embryos, and their HA titers were determined by the hemagglutination assay. The rg-A/American wigeon/South Carolina/22-000345-001/2021 (rgH5N1) virus is a reverse genetic-derived virus that contains six internal genes from the PR8 H1N1 virus and surface NA and modified HA genes from the clade 2.3.4.4b A/American wigeon/South Carolina/22-000345-001/2021 (H5N1) virus. The modified HA gene has only one basic amino acid at the HA cleavage site, and its protein sequence has three amino acid differences (P101R, L131Q, and T211I) when compared with that of the first bovine isolate A/cattle/Texas/56283/2024 (H5N1) virus. The human A/Cambodia/e0826360/2020 (H3N2) is a vaccine seed strain during the 2021–2022 flu season, while the A/Guangdong-Maonan/SWL1536/2019 (H1N1)pdm09 is a vaccine seed strain during the 2020–2021 flu season. The A/swine/Texas/4199-2/1998 (H3N2) is a cluster I H3N2 virus that was first detected in swine in the US in 1998 (40), while the A/swine/Kansas/11–110529/2011 (H3N2) belongs to the cluster IV H3N2 virus (41). There is no cross-reaction between these two H3N2 viruses (42). The A/swine/Kansas/12-156064/2012 (H1N2) (δ1 clade), which has the M gene from the A(H1N1)pdm09, is named H1N2 variant (swH1N2v) that has caused human infections in the US (43, 44). The H1N1 A/CA/04/2009 (CA09) was amplified on Madin–Darby canine kidney (MDCK) cells and used for the immunofluorescence assay (IFA).

### Nucleoprotein expression and purification

The NP gene of A/quail/Hong Kong/G1/1997 (H9N2) was cloned into the plasmid pET28a and verified by sequencing. The plasmid pET28a-NP was transformed into *E. coli* BL21 to express the NP protein. The transformed cells were cultured in 5 mL of Luria–Bertani (LB) medium containing 50 µg/mL kanamycin (Kan), shaking at 37°C overnight. Two milliliters of the preculture was then transferred into 200 mL fresh LB medium containing 50 µg/mL Kan and grown with shaking at 37°C until optical density (OD) values were measured at 600 nm reaching 0.8. The flask was placed on ice, and the *E. coli* was induced by adding 0.8 mM isopropyl β-D-1-thiogalactopyranoside (IPTG) and culturing at 16°C for 18 h. After the induction, the cells were harvested by centrifuging at 4,500 rpm for 30 min at 4°C and resuspended in 50 mL of lysis buffer (0.5 mol/L NaCl, 20 mmol/L Tris-HCl, pH 8.0). The suspended cells were sonicated for 30 min with an Ultrasonic (Fisher Scientific) on ice. The suspension was centrifuged at 12,000 rpm for 10 min to separate the cell debris from the lysate, and the supernatant fraction was analyzed by Western blot for protein expression. The protein was purified by HisPur Ni-NTA Resin (Thermo Scientific) according to the manufacturer’s instructions. The protein samples were then subjected to Amicon Ultra Centrifugal Filter (30 kDa MWCO) (Millipore Sigma). NP protein purity was analyzed by 10% sodium dodecyl sulfate-polyacrylamide gel electrophoresis (SDS-PAGE) and stained with the Coomassie Brilliant Blue R250.

### ELISA assay

The ELISA assay to detect bovine antibodies against IAV NP was optimized according to a previously published protocol (45). Briefly, Costar 96-well flat-bottom ELISA plates were coated with 10 ng/well of NP in 0.05 M carbonate–bicarbonate buffer (pH 9.6). Plates were washed three times with 300 µL/well of 0.1% phosphate-buffered saline-Tween 20 (PBST) and then blocked for 2 h at 37°C with 100 µL/well of 5% horse serum in PBS. Bovine serum samples were diluted 1:100 in PBS, and 100 µL/well was dispensed in duplicate. Positive HPAIV H5N1 serum collected from dairy cattle during the HPAIV outbreak and negative bovine serum samples were also diluted 1:100 in PBS to be used as controls. Plates were incubated for 1 h at 37°C and then washed with PBST three times. Horseradish peroxidase (HRP)-conjugated mouse anti-bovine IgG (Invitrogen) second antibody was diluted 1:100,000 in PBS and applied 100 µL/well and incubated for 1 h at 37°C. After washing the plates three times with PBST, the substrate (1-Step Ultra TMB-ELISA, Thermo Scientific) was added in the dark at 100 µL/well and incubated at room temperature for 10 min. The reaction was stopped with 100 µL/well of 2 M H_2_SO_4_. OD values were measured at 450 nm using FLUOstar Omega. To determine the cutoff value, three standard deviations to the corrected OD value of a panel of 10 negative-control sera diluted 1:100 were calculated. Serum samples were considered positive if corrected OD values were greater than or equal to the cutoff value and as negative if less than the cutoff value.

### Hemagglutination (HA) assay and hemagglutination inhibition (HI) assay

The HA assay was performed to determine HA titer of tested IAVs based on the standard protocol using 0.75% chicken red blood cells (RBCs). HI assay was conducted as described previously (46). Briefly, one volume of serum sample was treated with three volumes of receptor destroying enzyme (RDE) to remove nonspecific inhibitors of hemagglutination. The mixture was incubated in a 37°C water bath for 18–20 h, followed by 30 min incubation at 56°C water bath to inactivate the RDE. Heat-inactivated bovine serum samples were tested in duplicate and diluted twofold in PBS against four hemagglutination units (4 HAU) of the tested rgH5N1, H1N2v, seasonal H1N1, seasonal H3N2, and cluster I and VI H3N2 viruses in V-bottom plates with room temperature incubation for 30 min. Then, 50 µL of 0.75% chicken RBCs was added to the virus–serum mixture and incubated for another 30 min prior to result reading. The HI titer was the reciprocal of the last dilution of antiserum that completely inhibits hemagglutination.

### Validation of NP ELISA assay

To validate the developed NP ELISA assay, an IFA assay was performed to test randomly selected NP ELISA-positive and -negative bovine serum samples. Briefly, confluent MDCK cells on 96-well plates were infected with the H1N1 CA09 virus at a multiplicity of infection of 1 for 36 h, then were fixed with 100% methanol to permeabilize cells and preserve morphology for 30 min on ice. The fixed cells were rinsed with 1× PBS three times. Tested bovine serum samples (1:10 dilution) were added into each well and incubated at 37°C for 2 h, followed by staining with an anti-bovine IgG FITC-conjugate antibody (CJ-F-BOVG-AP-10ML, VMRD). Cells were washed with 1× PBST three times, and fluorescent signals were observed and captured using an EVOS M5000 microscope. To rule out potential cross-reactivity with IDV antibodies in bovine serum samples, three IDV-positive and one IDV-negative cattle serum samples collected from our previous studies (32) were tested by the NP ELISA assay as described above.

### Western blot

Equal amounts of purified NP protein were loaded into each well and run on 10% SDS-polyacrylamide gel (Invitrogen, Thermo Fisher Scientific). The protein was then transferred onto nitrocellulose membranes. The membrane was blocked with 5% dried skim milk in PBS overnight, followed by washing with 1 x PBST. The membrane was incubated with each tested serum sample (1:50 dilution) and incubated at room temperature for 1 h, then stained with an HRP-conjugated mouse anti-bovine IgG (H + L) second antibody (1:500 dilution, Invitrogen, MA5-16733). The membranes were developed with an enhanced chemiluminescent kit according to the manufacturer’s instructions (Invitrogen, Thermo Fisher Scientific). A serum sample (H3N2pc) collected from a calf infected with the TX98 H3N2 at 21 days post-infection was used as the positive control, and a bovine serum sample negative by HI and ELISA assays served as the negative control.

### Data analysis

The number of bovine serum samples tested by IAV NP ELISA from Jan 2023 to May 2024 was plotted by GraphPad Prism 10. Geographic distribution of bovine serum samples was analyzed and graphed by Tableau Public. The student *t*-test was used to analyze differences between HI-positive groups. A *P* ≤ 0.05 was considered statistically significant.

## Data Availability

Data presented in this study are available upon request. Materials are available upon request through the filing of material transfer agreements.

## EDITOR'S NOTE

A conflict of interest was identified after acceptance of this paper. The editor-in-chief provided a final review.

## SUPPLEMENTAL MATERIAL

Table S1 jvi.02138-24-s0001.docx Summary of HI-positive bovine serum samples.

ASM does not own the copyrights to Supplemental Material that may be linked to, or accessed through, an article. The authors have granted ASM a non-exclusive, world-wide license to publish the Supplemental Material files. Please contact the corresponding author directly for reuse.
