# Supplementary material for: Detection of antibodies against influenza A viruses in cattle
Source: J Virol. 2025 Mar 25;99(4):e02138-24. doi: 10.1128/jvi.02138-24 (PMC11998525; doi:10.1128/jvi.02138-24)
Supplement: Table S1 — Summary of HI-positive bovine serum samples. [file jvi.02138-24-s0001.docx]

**Supplemental Table 1: Summary of HI-positive bovine serum samples**

| **Positive ID** | **HI titer** | | | | |
| --- | --- | --- | --- | --- | --- |
|  | **swH1N2v** | **Seasonal huH1N1** | **Cluster IV swH3N2** | **Seasonal huH3N2** | **Cluster I swH3N2** |
| 5040-7 | 0 | 128 | 0 | 0 | 0 |
| 5906-22 | 64 | 32 | 0 | 0 | 0 |
| 31502-2 | 32 | n/d | n/d | n/d | n/d |
| 3010-51 | 0 | 64 | 0 | 0 | 0 |
| 7701-1 | 0 | 256 | 0 | 0 | 0 |
| 23096 | 0 | 256 | 0 | 0 | 0 |
| 22970 | 0 | 256 | 0 | 0 | 0 |
| 20062-2 | 0 | 32 | 0 | 0 | 0 |
| 19757 | 0 | 32 | 0 | 0 | 0 |
| 3803 | 0 | 32 | 0 | 0 | 0 |
| 3005 | 0 | 256 | 0 | 0 | 0 |
| 12688-39 | 0 | 64 | 0 | 0 | 0 |
| 11660 | 0 | 64 | 0 | 0 | 0 |
| 10874-1 | 0 | 128 | 0 | 0 | 0 |
| 14301-1 | 0 | 0 | 0 | 32 | 64 |
| 29916-9 | 0 | 0 | 32 | 0 | 0 |
| 29916-21 | 0 | 128 | 0 | 0 | 0 |
| 29794-1 | 0 | 128 | 0 | 0 | 0 |
| 2788-8 | 0 | 256 | 0 | 0 | 0 |
| 2636-1 | 0 | 256 | 0 | 0 | 0 |
| 1506-3 | 0 | 256 | 0 | 0 | 0 |
| 1477 | 0 | 256 | 0 | 0 | 0 |
| 1382-4 | 0 | 256 | 0 | 0 | 0 |
| 1326 | 0 | 256 | 0 | 0 | 0 |
| 7718-2 | 0 | 32 | 0 | 0 | 0 |
| 7749-1 | 0 | 32 | 0 | 0 | 0 |
| 7753-3 | 0 | 32 | 0 | 0 | 0 |
| 7753-7 | 0 | 32 | 0 | 0 | 0 |
| 3916 | 0 | 32 | 0 | 0 | 0 |
| 2280-4 | 0 | 0 | 0 | 32 | 32 |
| 4904-1 | 0 | 0 | 0 | 32 | 32 |
| 6658-6 | 0 | 128 | 0 | 32 | 32 |
| 8838 | 0 | 32 | 0 | 0 | 32 |
| 27003-3 | 0 | 128 | 0 | 0 | 0 |
| 27002-3 | 0 | 256 | 0 | 0 | 0 |
| 16756 | 0 | 256 | 0 | 0 | 0 |
| 2114-4 | 0 | 0 | 0 | 0 | 32 |
| 2280-3 | 0 | 0 | 0 | 0 | 32 |
| 5260 | 0 | 0 | 0 | 0 | 64 |
| 31494-1 | 0 | 0 | 0 | 0 | 32 |
| 2944-1 | 0 | 0 | 0 | 0 | 32 |
| 11228-1 | 0 | 0 | 0 | 0 | 64 |
| 15261-1 | 0 | 0 | 0 | 0 | 32 |
| 31788-7 | 0 | 0 | 0 | 32 | 0 |
| 2364-1 | 0 | 0 | 0 | 32 | 0 |

n/d: not done as the sample was used up
